# Novel Insights into the Interplay between Apoptosis and Autophagy

**DOI:** 10.1155/2012/317645

**Published:** 2012-03-15

**Authors:** Hidemi Rikiishi

**Affiliations:** Department of Microbiology and Immunology, Tohoku University Graduate School of Dentistry, 4-1 Seiryo-machi, Aoba-ku, Sendai 980-8575, Japan

## Abstract

For several decades, apoptosis has taken center stage as the principal mechanism of programmed cell death (type I cell death) in mammalian tissues. Autophagic cell death (type II) is characterized by the massive accumulation of autophagic vacuoles in the cytoplasm of cells. The autophagic process is activated as an adaptive response to a variety of extracellular and intracellular stresses, including nutrient deprivation, hormonal or therapeutic treatment, pathogenic infection, aggregated and misfolded proteins, and damaged organelles. Increasing evidence indicates that autophagy is associated with a number of pathological processes, including cancer. The regulation of autophagy in cancer cells is complex since it can enhance cancer cell survival in response to certain stresses, while it can also act to suppress the initiation of cancer growth. This paper focused on recent advances regarding autophagy in cancer and the techniques currently available to manipulate autophagy.

## 1. Introduction

Current cancer therapies are based on the surgical removal of solid tumor masses, usually combined with a series of chemical (chemotherapy) or physical (radiotherapy) treatments; however, chemotherapy has reached a plateau of efficacy as a treatment modality with the emergence of resistant tumors. Despite the wide variety of mechanisms, most new drugs are thought to ultimately induce apoptosis of tumor cells through mitochondrial and/or death-receptor pathways, although these pathways are often defective in cancer. More recently, other mechanisms of cell death have emerged as potential novel mechanisms for cancer therapies to induce cell death.

Macroautophagy (hereafter referred to autophagy) is a eukaryotic, evolutionarily conserved homeostatic process in which organelles and bulk proteins are turned over by lysosomal activity [1]. Autophagy and apoptosis may be interconnected and even simultaneously regulated by the same trigger in tumor cells. In periods of metabolic stress, autophagy provides ATP and other macromolecules as energy sources to enable cell survival; however, if the intensity or duration of metabolic stress is excessive, cells may progress to autophagic programmed cell death, which is distinct from apoptosis [2]. In contrast, whether autophagy contributes to the antitumor effect of chemotherapeutic drugs or to drug resistance is largely unknown. In addition, there is no current consensus on how to manipulate autophagy to improve clinical outcomes. This paper describes the role of autophagy with a particular focus on the roles of cytoplasmic organelles and presents newly recognized approaches to pharmacologically exploit these mechanisms for improved antitumor outcomes.

## 2. Autophagy Networks

### 2.1. Degradation System

Eukaryotic cells respond to changes in their environment and the intracellular milieu by altering their anabolic and catabolic pathways. As one of the responses, living organisms from yeast to humans are capable of eating parts of themselves in order to survive. The so-called autophagy is a self-degradative process which ensures the regular turnover of cellular components by sequestering damaged organelles and misfolded proteins, targeting them for lysosomal degradation [3]. While the ubiquitin-proteasomal system is generally used for the degradation of short-lived proteins, autophagy degrades and recycles long-lived proteins and organelles. After its discovery, autophagy was considered a kind of disposal mechanism aimed at recycling of cellular components [4], but now autophagy is implicated in more diverse physiological processes such as development, proliferation, remodeling, aging, tumor suppression, neurodegeneration, antigen presentation, innate immunity, regulation of organismal lifespan, and cell death [5]. In most of these situations, autophagy has both beneficial and harmful effects on cell functions. One aspect of this complexity probably reflects the dual role of autophagy, which is both cell-protective and -destructive. Autophagy can be a survival response in situations of stress, allowing the elimination of toxic metabolites, intracellular pathogens, and damaged proteins and organelles and providing energy and amino acids necessary for vital functions during metabolic stress; however, in some sense the induction of excessive autophagy can lead to cell death. In addition, autophagy provides signals for removal of apoptotic cells and genomic stability [6]. Hence, autophagy can be generally considered as a cell protector against various types of injuries or continuous cellular wear and tear and is expected to play a protective role in diverse types of cellular stress. Paradoxically, autophagy can also lead to a form of nonapoptotic cell death, which is called type II programmed cell death [7]. Thus, autophagy could either promote cell death or protect cells from diverse types of injuries depending on the cellular and environmental context.

### 2.2. Signaling System

The serine/threonine kinase, mammalian target of rapamycin (mTOR), is a major negative regulator of autophagy. The phosphoinositide 3-kinase- (PI3K-) activated serine/threonine kinase Akt phosphorylates the mTOR repressor TSC2 (tuberous sclerosis complex 2), thus leading to activation of mTOR and subsequent blockade of expression and function of autophagy-inducing Atg proteins. In addition to Akt, one of the main mTOR regulators is AMP-activated protein kinase (AMPK), a principal energy-sensing intracellular enzyme activated in various cellular and environmental stress conditions. In response to increases in AMP/ATP ratio, AMPK preserves energy by switching off ATP-requiring processes, while switching on ATP-generating catabolic pathways. AMPK maintains energy homeostasis by inducing autophagy and blocking protein synthesis and cell proliferation mainly through phosphorylation of its downstream target Raptor and consequent inhibition of mTOR. In addition to a major role of the AMPK/mTOR pathway in regulation of intracellular and whole body metabolism, recent findings point to its potential involvement in controlling proliferation, survival, and death of cancer cells [8]. Namely, pharmacological activation or overexpression of AMPK in a variety of cancer cell types caused an mTOR inhibition-associated cell cycle arrest and apoptotic death both *in vitro* and *in vivo* [9]. On the other hand, AMPK activity in certain conditions protected normal and cancer cells from metabolic stress and/or chemotherapy-induced apoptosis [10]. Autophagic digestion of intracellular proteins triggered by mTOR-downregulation in tumor cells seems to play an important role both in AMPK-mediated cytotoxicity and cytoprotection [11, 12], the final outcome possibly depending on the nature of the cytotoxic stimulus. The physiological role of autophagy in the mammalian system has been shown in several *in vivo* animal models. Disruption of the autophagic process leads to failure of cavitation during embryogenesis or accumulation of abnormal organelles such as mitochondria or endoplasmic reticulum (ER) in adult tissues [13, 14]. In addition to the physiological roles, dysregulated autophagy has been suggested to play pathogenetic roles in a variety of disease processes, particularly when cellular stress is increased, which is probably because of the accumulation of damaged molecules and organelles. It has been suggested that the role of autophagy in degenerative changes is associated with ageing or diverse degenerative disorders [15].

## 3. Programmed Cell Death

The term programmed cell death refers to controlled or regulated forms of cell death associated with a series of biochemical and morphological changes. Programmed cell death is an evolutionarily conserved process to decide cell fate, which has therefore been drawing increasing attention in cancer treatment. Programmed cell death can be divided into several categories: type I (apoptosis), type II (autophagic cell death), and others (necrosis, senescence, and mitotic catastrophe).

### 3.1. Type I Programmed Cell Death

In type I, the typical morphology of apoptosis is largely the end result of caspase-mediated destruction of the cellular structure [16]. There are two core pathways inducing apoptosis: the extrinsic and intrinsic pathways. The extrinsic pathway is triggered by the Fas death receptor (DR), which is mainly dependent on the initiation of the combination between FasL and Fas. The intrinsic pathway is another process leading to apoptosis, in which mitochondria play a central role. When cells sense extracellular stimuli or intracellular signals, the outer mitochondrial membrane becomes permeable, releasing cytochrome *c*. The ER, lysosomes, and the *trans*-Golgi-network also play important roles [17]. Each organelle possesses sensors that detect specific alterations, locally activate signal transduction pathways, and emit signals that ensure interorganellar cross-talk. It is often assumed that apoptosis is initiated if the damage is too severe to be repaired, or if the time-scale for complete repair is too long.

### 3.2. Type II Programmed Cell Death

Autophagy has been associated with a particular mode of cell death that is characterized by the appearance of double- or multiple-membrane cytoplasmic vesicles engulfing bulk cytoplasm and/or cytoplasmic organelles such as mitochondria and ER; however, many studies failed to demonstrate a causal link between autophagy and cell death. Autophagy is probably not an executor of cell death *per se*, but it is a required process in certain settings in combination with other prodeath signals. Cells undergoing autophagic cell death were engulfed by human macrophages as well as vital cells [18]. To distinguish it from apoptosis (type I cell death), this has been termed autophagic or type II cell death [19]. The machineries for apoptosis, autophagy, and necrosis (type III cell death) are interconnected and somehow coordinated between the two. For example, the elimination of damaged mitochondria by autophagy would prevent the release of proapoptotic substances from mitochondria, thus preventing apoptosis. In the absence of such clean up, the release of molecules like cytochrome *c* and apoptosis-inducing factor (AIF) from damaged mitochondria would lead to apoptosis and in the case of extreme damage and ATP depletion, to necrotic cell death.

## 4. Autophagy in Cancer

There is an accumulation of evidence that highlights the important function of autophagy in cancer. Although it is still controversial as to whether autophagy kills cancer cells or sustains their survival under stressful conditions, more reports provide data to support that autophagy promotes cancer cell survival after chemotherapy or radiation therapy [20]. The high rate of autophagy observed in undifferentiated colon cancer cells is compatible with the expression of Beclin 1 and PTEN (phosphatase and tensin homolog) in these cells. Cancer cells are frequently exposed to inherent metabolic stress owing to hypoxia and lack of nutrient supplies. Hypoxia-inducible factor 1*α* (HIF-1*α*), a key transcription factor encoding a plethora of genes responsible for altered metabolism, angiogenesis, invasion, metastasis, or therapy-resistance in hypoxic tumors [21], is a positive regulator of autophagy. Small-interfering-RNA- (siRNA-) mediated depletion of Atg proteins sensitizes cancer cells to radio- and chemotherapy, and the autophagy inhibitors 3-methyladenine (3-MA) and bafilomycin A1 cause radiosensitization of malignant glioma cells [20]. Thus, cancer was one of the first diseases genetically linked to impaired autophagy.

The tumor-suppressive role of autophagy was first shown in mice heterozygous for the Beclin 1 autophagy protein. These mice (*beclin *1^+/−^) showed reduced autophagy and increased cellular proliferation, which translated into increased incidence of spontaneous malignancies, such as lymphomas, lung, and liver cancers [22]. Incidentally, *beclin *1^−/−^ mutant mice died early in embryonic development. Liang et al. reported *beclin 1* as a potential tumor suppressor gene, the expression of which is frequently decreased in human breast epithelial carcinoma cell lines and tissue compared to the higher level in normal tissue [23]. Low expression of Beclin 1 in 115 node-positive colon cancer specimens was associated with a significantly worse 5-year overall survival (47% versus 67%) [24]. The PI3K/Akt/mTOR axis plays a decisive role in the negative regulation of autophagy, and the constitutive activation of this pathway has been implicated in many human cancers [25]. Autophagy is thought to mediate Sqstm1/p62 elimination and to suppress tumorigenesis; however, defective autophagy allows overexpression of p62 that promotes tumorigenesis. The sustained p62 overexpression in tumor cells with defective autophagy appears to dysregulate NF-*κ*B signaling, and altered NF-*κ*B regulation resulting from p62 overexpression is proposed to be a primary mechanism for enhancing tumorigenesis [26]. Persistent DNA damage induced by the increased levels of reactive oxygen species (ROS), which are associated with the accumulated mutant proteins and dysfunctional mitochondria found in autophagy-deficient tissues, is also proposed as a causative link between autophagy inhibition and enhanced tumor formation [27]. Thus, the inability of autophagy-defective tumor cells to eliminate p62 contributes to oxidative stress and likely to DNA damage. These observations suggest that autophagy is important for tumor suppression.

Ultimately, autophagy in cancer has different effects in different ways in different cancer cell lines at different stages of tumorigenesis and progression. The early stages of tumor development require cancer cells to undergo a higher level of protein synthesis than protein degradation for the tumor to grow. Thus, autophagy probably functions to prevent cancer initially, but once a tumor develops, the cancer cells utilize autophagy for their own cytoprotection. Elucidating this complex physiological process is crucial for development of anticancer therapies with maximum efficacy and minimum drug resistance.

## 5. Cross-Talk between Apoptosis and Autophagy in Cancer

Expanding our knowledge of the molecular cross-talk among pathways that regulate tumor cell death is crucial in guiding the successful design of future anticancer therapeutics. In particular, apoptosis and autophagy can act as partners to induce cell death in a coordinated or cooperative fashion. Autophagy proteins can also play a role in cellular events that occur during apoptosis. For example, Atg5 may be an independent key player in both apoptosis and autophagy. The low levels of Atg5 cleavage product may have significant effects on apoptosis, but not the intact Atg5 that participates in autophagy [28]. Bcl-2 phosphorylation may not only be a mechanism for regulating apoptosis and a mechanism for regulating autophagy, but perhaps also a mechanism for regulating the switch between the two pathways [29]. JNK (c-Jun NH_2_-terminal kinase) is able to trigger autophagy by targeting Bcl-2/Bcl-xL proteins and abrogating their binding to Beclin 1. Recently, Beclin 1 has been shown to be among the substrates of death-associated protein kinase (DAPK), a proapoptotic serine/threonine kinase, and its phosphorylation reduces its binding to the Bcl-2 family members, thus suggesting a possible mechanism by which DAPK may also induce autophagy [30]. A complex role in the regulation of autophagy is played by p53, one of the most important tumor suppressor proteins. In fact, p53 regulates autophagy both in a positive and in a negative fashion, depending on its subcellular localization [31]. p53 and DRAM (damage-regulated autophagy modulator) can induce accumulation of autophagosomes. In addition, p53 affects autophagy by modulating signaling through the mTOR nutrient-sensing kinase which controls autophagy at the initiation stage [32]. Inhibition of autophagy leads in most cases to an increase susceptibility to apoptotic stimuli. Moreover, a growing number of proteins that play a negative regulatory role in both events have been identified [33]. Autophagy and apoptosis may be triggered by common upstream signals. Recent reports have shown that Akt inhibits apoptosis by phosphorylation of the Bcl-2 protein family member, Bad, allowing for cell survival. In addition, activation of the PI3K/Akt/mTOR pathway can cause inhibitory effects of Akt on apoptosis and mTOR on autophagy and enhance survival capacity in neoplastic cells [34].

In addition, several Atg proteins, including Atg5 and Beclin 1 (Atg6), can be cleaved by calpain or caspases that are activated during apoptosis [35]. Caspase-dependent cleavage of Beclin 1 occurred in HeLa cells treated with a death receptor ligand [36]. Chemotherapy-induced apoptosis suppresses autophagy at the execution stage following cytochrome *c* release, at least in part through caspase 8-mediated cleavage of Beclin 1 at Asp^133^ and Asp^146^ [37]. Caspase 3 cleaved Beclin 1 at Asp^149^ in apoptosis induced by Bax overexpression [38]. Caspase-9 can also cleave Beclin 1, thereby destroying its proautophagic activity [39]. Moreover, the C-terminal fragment of Beclin 1 that results from this cleavage acquires a new function, which can amplify mitochondrion-mediated apoptosis. These findings provide important insights into the molecular cross-talk between autophagy and apoptosis. Apoptosis can begin with autophagy, which can end with apoptosis. Some links between the two types of cell death are indicated via mitochondria. Induction of mitochondrial membrane permeabilization at a low level, below the threshold required for induction of apoptosis, results in sequestering damaged mitochondria in autophagic vacuoles. When mitochondrial membrane permeabilization is sufficiently high to sustain the active execution of cell death, apoptosis is induced [40]. Therefore, it is likely that induction of apoptotic or autophagic cell death may depend on the level of mitochondrial membrane permeabilization.

## 6. ER and Autophagy

ER is an essential intracellular organelle providing apparatus for synthesizing nascent proteins as well as their further modification and correct folding, such as the formation of N-linked glycans and disulphide bonds. Disruption of any of these processes, such as with glucose depletion that prevents the proper glycosylation of proteins or with alterations in calcium homeostasis such that calcium-dependent chaperones cannot function properly, leads to the accumulation of misfolded proteins that triggers ER stress. ER stress has been implicated in different stages of tumor development. The ER has by now been established as an essential site for the regulation of apoptotic pathways and has recently been recognized as an important component of autophagic signaling. ER stress appears to signal autophagy by pathways that all depend on eIF2*α* (eukaryotic initiation factor 2*α*). How eIF2*α* regulates autophagy is presently unknown, but the induction of Atg12 expression via ATF4 (activating transcription factor 4) is likely to participate in this process. In cancer cells, when the amount of unfolded or misfolded proteins exceeds the capacity of the proteasome-mediated degradation system, autophagy is triggered to remove these proteins.

Bcl-2 has been mainly studied in the context of cell death, but it is also known to carry out other functions in different cellular processes, such as cell cycle progression, glucose homeostasis, transcriptional repression by p53, and autophagy. Beclin 1 has been identified as a novel BH3- (Bcl-2-homology-3-) only protein. Beclin 1 induces autophagy by promoting autophagosome formation when in complex with hVps34 (vacuolar protein sorting 34)/class III PI3K. Beclin 1-Bcl-2 (Bcl-xL) complexes normally inhibit autophagy, which points to organelle-specific regulation of autophagy [41]. Beclin 1 binding with Bcl-2 has been suggested to interfere with the ability of Beclin 1 to form a complex with hVps34/PI3K, thus resulting in a loss of Beclin 1-associated PI3K autophagy-inducing activity. The interaction between Beclin 1 and Bcl-2 is abolished when key residues within the BH3 domain of Beclin 1 or the BH3-binding groove of Bcl-2 are mutated. ER-localized Bcl-2, but not mitochondria-localized Bcl-2, has been shown to be important for the inhibition of autophagy [42]. The ER functions to synthesize proteins that store high intracellular calcium that can activate calcium-dependent chaperone proteins that assist in protein folding. The autophagy inhibition by Bcl-2 is evident only when Bcl-2 resides in the ER, where it has been suggested to regulate cellular Ca^2+^ homeostasis. Ca^2+^ regulates autophagy via a signaling pathway involving AMPK and mTOR, and ER-located Bcl-2 effectively inhibits this pathway. ER-located Bcl-2 inhibits autophagy induced by Ca^2+^ mobilizing agents by regulating the Ca^2+^ homeostasis in a Beclin 1-independent manner [43].

## 7. Golgi and Autophagy

Eukaryotic cells possess an internal endomembrane system that compartmentalizes the cell for various different cellular functions. The main components of this membrane system are the ER, Golgi complex, vesicles, plasma membrane, vacuole/lysosome, and nuclear envelope. Autophagy displays unique membrane dynamics, distinct from classical membrane trafficking. The Golgi complex is required for double-membrane cytoplasm to vacuole targeting vesicle and autophagosome formation. A number of Rab GTPases which regulate secretory and endocytic membrane traffic have been shown to play either critical or accessory roles in autophagy. Rab33B, a member of the Rab small GTPase family, which was originally described as a Golgi-resident protein involved in Golgi-to-ER transport [44], directly interacts with an autophagosome precursor marker, Atg16L, in a GTP-dependent manner, and the activation and inactivation of Rab33B modulate autophagy. Based on these results, the possible role of the interaction between Rab33B and Atg16L was suggested in autophagosome formation [45]. The autophagosomes are not formed by Golgi-derived vesicles/membranes alone because single-membrane structures, the so-called isolation membranes, and autophagosome membranes are composed of membranes derived from rough ER. ER and Golgi complex-derived vesicles could be delivered to the lysosome/vacuole by a pathway that is different from canonical autophagy. Under certain conditions, these two organelles might not require sequestration by a double membrane for delivery and subsequent degradation. Furthermore, it has been reported that one of the Atg proteins, Atg9, a membrane protein with unknown function, shuttled between the* trans*-Golgi network and late endosomes. Bax-interacting factor 1 (Bif-1) binds the proapoptotic Bcl-2 family protein Bax. Bif-1 overexpression promotes Bax activation and apoptosis. Bif-1 also regulates the fission of Golgi membranes and the trafficking of Atg9 from the Golgi complex to autophagosomes during starvation [46]. Therefore, isolation membranes and autophagosome membranes may be made up of membranes from several different sources, including the Golgi; however, there is little evidence that the Golgi complex contributes directly to the formation of the autophagic membrane.

## 8. Mitochondria and Autophagy

Mitochondria are key players in several cancer cellular functions, including growth, division, energy metabolism, and apoptosis, and play a central role in cell survival and death. Mitochondria also play an important role in the formation of autophagosome and its subsequent docking and fusion with lysosome and could contribute to the increased autophagy and autophagic flux in metastatic cancer. The ability of tumor mitochondria to increase autophagy may help cancer cells to fulfill high anabolic needs during rapid growth; however, mitochondrial functions (such as respiration) can block autophagic cell death [47]. Autophagy often occurs when the mitochondria fail to maintain ATP levels, during starvation, or when the mitochondria are damaged. A process known as mitophagy selectively eliminates the damaged mitochondria. Furthermore, blocking mitochondria-mediated apoptosis through the elimination of mouse *Bax *and *Bak *(*Bak1*) expression promotes autophagy-induced cell death. Arsenic-trioxide- (As_2_O_3_-) induced cell death in human malignant glioma cell lines was accompanied by involvement of an autophagy-specific marker, LC3, and damage to mitochondrial membrane integrity. Arsenic trioxide induces the mitochondrial localization of the pro-cell-death Bcl-2 family member BNIP3 (Bcl-2/adenovirus E1B 19-kDa protein-interacting protein 3), which determines the on/off state of the mitochondrial permeability transition (MPT) pore [48]. The reciprocal relationship between mitochondrial metabolism and the activity of the rapamycin-sensitive pathway has been described before [49]. Mitochondria dysfunction leads to inhibition of mTOR, and inactivation of p70S6K (ribosomal protein S6 kinase) results in dephosphorylation of Bad, potentially leading to increased interaction between Bad and Bcl-xL or Bcl-2, to mitochondrial damage and cell death [50]. In addition, the promotion of MPT also contributed to increased autophagy. These results suggested that mitochondria from a highly metastatic breast cancer cell line can promote homeostatic autophagy of cancer by opening low-conductance MPT pores. The activation of autophagy by tumor mitochondria was reduced by cyclosporine A, which is an inhibitor that blocks the low-conductance state of MPT pores [47]. ROS could be the mediator leading to the loss of mitochondria membrane potential, possibly by triggering the opening of the MPT pore that subsequently leads to cell death. ROS were recently shown to activate starvation-induced autophagy, antibacterial autophagy, and autophagic cell death. Short-chain fatty acids were shown to induce ROS production, which led to AMPK activation and consequential mTOR inhibition [51]. Current findings implicate ROS in the regulation of autophagy through distinct mechanisms, depending on the cell type and stimulation conditions; conversely, autophagy can also suppress ROS production.

## 9. Experimental Manipulation of Autophagy

For a better understanding of the regulation of autophagy, many studies have used chemical modulators of autophagy: inhibitors and inducers. The different modulators are an important tool in studying the functions of autophagy, although it is difficult to define the mechanism closely. Appropriate modification of autophagy, that is, inhibition of cytoprotective autophagy or promotion of cyto-killing autophagy could mitigate the cytotoxicity caused by anticancer therapy in tumor cells.

### 9.1. ProAutophagics (Table 1)

#### 9.1.1. Beclin 1

The mechanism by which a small molecule Bcl-2/Bcl-xL antagonist *ABT-737* can potentiate autophagy may be related to its ability to competitively disrupt the binding of Bcl-2/Bcl-xL to the autophagic protein Beclin 1 (Atg6) [52]. Treatment with *fenretinide* (RT-101), a synthetic derivative of retinoic acid, resulted in an increase in Beclin 1 expression, the conversion to the form LC3-II form, and its shift from diffuse to punctate staining and finally an increase in lysosomes/autophagosomes [53]. In addition, HIF-1*α* is required for *fenretinide*-induced protective autophagy under hypoxia. The inositol 1,4,5-trisphosphate receptor (IP_3_R) antagonist *xestospongin B* induces autophagy by disrupting a molecular complex formed by IP_3_R and Beclin 1. *Xestospongin B*-induced autophagy was inhibited by overexpression of the IP_3_R ligand-binding domain, which co-immunoprecipitated with Beclin 1 [54]. Autophagy can be generally induced via several distinct pathways, such as inhibition of mTOR or activation of Beclin 1. *Okadaic acid*, a protein phosphatase 2A inhibitor, increased both the mTOR and Beclin 1 pathways simultaneously, which suggests that autophagy in *okadaic acid*-treated neurons is induced mainly via the Beclin 1 pathway, and less so via mTOR inhibition [55].

#### 9.1.2. DNA Damage

Exogenous environmental agents such as *ultraviolet* (UV) and *ionizing radiation*, genotoxic chemicals, and endogenous byproducts of metabolism, including *ROS*, can cause alterations in DNA structure (DNA damage). DNA damage induces autophagy, but its role in the DNA damage response is still unclear. Recent reports using the DNA-damaging agents, *camptothecin*, *etoposide* (VP-16), *tomozolomide*, and *p-anilioaniline* (*p*-aminodiphenylamine), demonstrate that cells, in addition to initiating cell cycle arrest, also initiate autophagy. p53 is a central regulator of apoptosis induced by DNA damage. Interestingly, p53 is a bidirectional regulator of autophagy. p53 activation also leads to upregulation of the PTEN, an inhibitor of the PI3K/Akt signaling pathway, and TSC2, at the transcriptional level, which may contribute to the long-term suppression of mTOR [56].

#### 9.1.3. ER Stressors

 Autophagy was induced by chemicals, such as *A23187*, *tunicamycin*, *thapsigargin*, and *brefeldin A* that cause ER stress [57]. The calcium ionophore *A23187* induced the expression of BiP/GRP78 protein, which is an ER stress marker protein. GRP78, through maintenance of ER structure and homeostasis, facilitates autophagy. Disturbances in normal ER processes lead to an evolutionarily conserved cell stress response, referred to as the unfolded protein response (UPR). *Tunicamycin*, an inhibitor of N-glycosylation, is a potent stimulator of ER stress. The link between *tunicamycin*-induced ER stress and autophagy was the PERK/elF2*α* pathway. *Thapsigargin*, an inhibitor of the ER calcium transporters, generates Ca^2+^-store depletion within the ER and simultaneously increases the Ca^2+^ level in the cytosol. *Brefeldin A* (an inhibitor of vesicle transport between the ER and Golgi) can increase the expression of both glucose-regulated proteins GRP78 and 94, which disrupt some functions of the ER. The inhibition of the 26S proteasome by the dipeptide boronic acid *bortezomib* (velcade) leads to the accumulation of misfolded proteins, resulting in ER stress followed by a coordinated cellular UPR response [58]. Induction of autophagy by *bortezomib* is dependent on the proteasomal stabilisation of ATF4 and upregulation of LC3B by ATF4. *Sorafenib* (nexavar), a potent multikinase inhibitor, inhibited phosphorylation of Stat3 and expression of cyclins, D and E, and exerted significant antitumor activity through inhibition of mTOR signaling. In addition, *sorafenib* induced autophagy in human hepatocellular carcinoma cells through mechanisms that involved ER stress and was independent of the MEK1/2-ERK1/2 pathway [59].

#### 9.1.4. Farnesyltransferase Inhibitors

 Farnesylation is a posttranslational modification of proteins in which farnesyltransferase catalyzes the attachment of the isoprenoid group from farnesyl pyrophosphate to a cysteine residue at the C-terminus of proteins. The farnesyltransferase inhibitors *manumycin A*, *FTI-276*, and *lonafarnib* (sarasar) induced autophagy in human cancer cell lines [60]. Treatment with a combination of oridonin and *manumycin A* (a natural product of *Streptomyces parvulus*) downregulated phosphorylation of Akt, downstream of PI3K. Oridonin triggers apoptosis of cancer cells and is partially effective through mitochondrial depolarization. *Lonafarnib* decreased phosphorylation of mTOR and S6 kinase, which is downstream of mTOR, in a dose-dependent manner. *FTI-276* is a CAAX peptidomimetic of the carboxyl terminal of Ras proteins. *FTI-276* was identified as a highly selective suppressor of Ras-dependent oncogenicity, and *FTI-276* inhibited Akt phosphorylation.

#### 9.1.5. Golgi-Associated Agents

*Brefeldin A* inhibits the activation and membrane-binding properties of most ADP-ribosylation factors and causes the redistribution of Golgi proteins into the ER. *Brefeldin A* leads to dramatic changes in the structure of the Golgi apparatus causing the conversion of the staked cisternae into vesicular/cisternal remnants. *Brefeldin A* even when acting alone, increases the volume fraction of autophagic vacuoles. The destabilization of the Golgi apparatus by *brefeldin A* interrupts the intracellular trafficking of N-linked glycoproteins along the secretory pathway [61]. Treatment of cells with *paclitaxel* (taxol) results in polymerization of microtubules. After incubation with *paclitaxel*, the Golgi apparatus is fragmented and is conspicuously present in areas of the cytoplasm enriched in microtubules. *Paclitaxel* treatment could lead to the formation of acidic vesicular organelles, the induction of Atg5, Beclin 1, and LC3 expressions, and an increase in punctate fluorescent signals in A549 cells pretransfected with GFP-tagged LC3 [62].

#### 9.1.6. HDAC Inhibitors

Histone acetylation is mediated by histone acetyltransferases and deacetylases (HDACs), which influence chromatin dynamics, protein turnover, and the DNA damage response. The HDAC inhibitor *SAHA* (suberoylanilide hydroxamic acid, vorinostat) can induce both caspase-dependent apoptosis and caspase-independent autophagic cell death, so it has clear therapeutic implications [63]. *SAHA* induced autophagy through downregulation of Akt/mTOR signaling and induction of the ER stress response. Moreover, *SAHA* treatment upregulated expression of Beclin 1 and Atg7 and promoted formation of the Atg5-Atg12 conjugate [64]. *Valproic acid* was identified as a potent selective histone deacetylase inhibitor, which induces autophagy in glioma cells. *Valproic acid* is capable of producing elevated levels of mitochondrial ROS in glioma cells. ROS production results in autophagosome development and autolysosomal degradation [65]. *FK228* (romidepsin) is a unique cyclic peptide and is among the most potent inhibitors of both Class I and II HDACs. *FK228* converted LC3-I to LC3-II and induced localization of LC3 to autophagosomes. *FK228*-mediated autophagy in rhabdomyosarcoma cells coincided with nuclear translocation of AIF, while knockdown of AIF abrogated autophagy following *FK228* exposure [66].

#### 9.1.7. Mitochondrial Agents

An essential trace element, *selenite*, is preferentially cytotoxic to various human glioma cells over normal astrocytes via autophagic cell death. Before *selenite*-induced cell death in glioma cells, disruption of the mitochondrial cristae, loss of mitochondrial membrane potential, and subsequent entrapment of disorganized mitochondria within autophagosomes or autophagolysosomes, along with degradation of mitochondrial proteins, were noted, showing that *selenite* induces autophagy in which mitochondria serve as the main target [7]. The phytoalexin *resveratrol* (trans-3,5,4′-trihydroxystilbene), a defensive substance produced by plants in response to infection by pathogenic microorganisms, displays a wide range of biological effects in mammalian cells. *Resveratrol* induced autophagy through any of numerous mechanisms that involve activation of mitochondria, downregulation of cell survival proteins, inhibition of cell survival kinases, and survival transcription factors [67].

#### 9.1.8. mTOR Inhibitors

*Rapamycin* (sirolimus) and its analogues (such as *CCI-779*, *RAD001*, and *AP23573*) inhibit mTOR (mTORC1), the kinase that normally suppresses both apoptosis and autophagy. *Rapamycin* activates the autophagic process, and silencing of mTOR with siRNA increases the inhibitory effect of *rapamycin* on tumor cell viability by stimulating autophagy [68]. *Berberine*, a small molecule derived from *Coptidis rhizome*, can induce both autophagy and apoptosis in hepatocellular carcinoma cells. *Berberine* may also induce autophagic cell death in HepG2 and MHCC97-L cells through activation of Beclin 1 and inhibition of the mTOR-signaling pathway by suppressing the activity of Akt and upregulating p38 MAPK signaling [69]. Treatment with *torin1* significantly enhanced lysosomal accumulation of mTOR and Raptor. *Torin1* inhibits mTOR by directly competing with ATP for binding to the kinase domain. Thus, *torin1* inhibits both mTORC1 and mTORC2 [70]. The small molecule *Ku-0063794*, which inhibits both mTORC1 and mTORC2 with an IC_50_, induced a much greater dephosphorylation of the mTORC1 substrate 4E-BP1 (eukaryotic initiation factor 4E-binding protein 1) than *rapamycin* [71]. *PM02734* (elisidepsin) causes cell death by a complex mechanism that involves increased autophagosome content, due for the most part to impairment of autophagic flux, inhibition of the Akt/mTOR pathway, and activation of DAPK. This unique mechanism of action justifies the continued development of this agent for the treatment of lung cancer [72].

#### 9.1.9. Pathogens

 Autophagy has recently emerged as an important mechanism for controlling intracellular pathogens. A variety of different bacteria, including *Mycobacteria*, *Salmonella*, *Shigella*, and *Listeria*, are recognized by autophagy, yet the specific signals that mediate recognition of intracellular pathogens by the autophagy machinery have not been defined thus far. It is currently thought that ubiquitin associated with intracellular pathogens promotes targeted autophagosome formation and pathogen destruction. In addition, p62 and NDP52 mediate different pathways of selective autophagy for *Shigella *and *Listeria* and provide novel insight into the mechanisms by which adaptor proteins target bacteria to autophagy [73]. Infection with *Herpes simplex virus type 1* (*HSV-1*) provoked autophagy under conditions that inhibited viral gene expression. The induction of autophagy occurs very early after infection with *HSV-1*, and *de novo *protein synthesis is not required for the response [74]. Autophagy can be induced by the presence of foreign DNA within cells. The interferon-inducible, dsRNA-dependent protein kinase R (PKR) plays an important role in innate immunity against viral infections. PKR activation leads to phosphorylation of eIF2*α* and a subsequent shutdown of host and viral protein synthesis and viral replication. PKR and eIF2*α* phosphorylation regulate another fundamental cellular process, the lysosomal degradation pathway of autophagy.

#### 9.1.10. Proteasome Inhibitors

 The ubiquitin-proteasome system and lysosome-dependent autophagy are two major intracellular pathways for protein degradation. The ubiquitin-proteasome pathway is a novel therapeutic target for cancer treatment. Inhibition of proteasome function leads to the accumulation of polyubiquitinated proteins and likely causes the accumulation of misfolded proteins in the ER due to the blockage of ER-associated degradation. Thus, proteasome inhibitors activate autophagy via ER stress. Proteasome inhibitors have been potent anticancer agents for various cancers. Proteasome inhibitor *MG-132* increased the protein expression of LC3-I and -II in a time-dependent manner. 3-MA significantly abolished the formation of LC3^+^ autophagic vacuoles and the expression of LC3-II, but not LC3-I, induced by *MG-132* [75]. A new class of proteasome inhibitors such as *glidobactin A* treatment also induced autophagy as judged by the presence of the lipidated form of LC3 and autophagosomes [76]. The eukaryotic 20S proteasome contains three catalytic subunits (*β*1, *β*2, and *β*5) conferring caspase-like, trypsin-like, and chymotrypsin-like proteolytic activities, respectively. *Glidobactin A* blocked the chymotrypsin-like activity irreversibly at low concentrations, whereas the trypsin-like activity was less sensitive and the caspase-like activity was not inhibited at the concentrations tested (up to 20 *μ*M).

### 9.2. AntiAutophagics (Table 2)

#### 9.2.1. Atg Proteins

The regulation of the autophagic and apoptotic response has been shown to require components that are involved in both processes. *Caspases*, a family of cystinyl aspartate-requiring proteases, play a central role in apoptosis, a well-studied pathway of programmed cell death. *Calpain* is a calcium-dependent intracellular cysteine protease that plays a crucial role in the regulation of cell spreading, cell migration, programmed cell death, and cell cycle progression. The majority of human Atg proteins can be cleaved by *caspases* and *calpains*, which are activated in some apoptotic paradigms [77]. For example, Atg3 is cleaved by *caspase-3*, -*6,* and -*8*, while Atg9, Atg7, and Atg4 homologues can be cleaved by *caspase-3*. Beclin 1 (Atg6) is a substrate of *caspase-3* with two cleavage sites at positions 124 and 149, respectively. Cleavage of Beclin 1 was also seen in apoptosis of HeLa cells induced by staurosporine and TRAIL. The cleavage of Beclin 1 resulted in abrogation of the interaction between Bcl-2 with Beclin 1, which could be blocked by z-VAD-fmk. Atg5 is a gene product required for the formation of autophagosomes. *Calpains-1* and -*2* activation and Atg5 cleavage are general phenomena in apoptotic cells. Truncated Atg5 translocated from the cytosol to mitochondria, associated with the antiapoptotic molecule Bcl-xL, and triggered cytochrome *c* release and *caspase* activation. *Caspase* cleavage of autophagy-related proteins can affect the autophagic process.

#### 9.2.2. Autophagosome Formation

*Verteporfin* (visudyne) is a benzoporphyrin derivative used in photodynamic therapy, and *verteporfin* inhibited drug- and starvation-induced autophagic degradation and the sequestration of cytoplasmic materials into autophagosomes [78]. *Verteporfin* inhibited autophagy stimulated by serum starvation, a physiological stimulus, and by rapamycin, the chemical inhibitor of mTORC1. *Verteporfin* binds to the membrane of expanding phagophores or to a factor involved in phagophore expansion and prevents expanding phagophores from adopting their characteristic cup shape, so they are unable to capture cytoplasmic cargo and only form empty single-membrane vesicles. *3-Methyladenine* (*3-MA*), as an autophagy inhibitor, was first discovered via screening of purine-related substances using isolated hepatocytes from starved rats. *3-MA*, which interferes with the formation of autophagosomes in mammalian cells via inhibition of the class III PI3K activity that controls the autophagic pathway [79], has been widely used in studies on autophagy. *3-MA* has a dual role in modulation of autophagy; although it is capable of suppressing autophagy induced by starvation, its prolonged treatment in full medium induces autophagy. Under specific treatment conditions, *3-MA* acts similar to rapamycin, a well-established autophagy inducer via suppression of mTOR function.

#### 9.2.3. Bcl-2 Family

Apoptosis and autophagy are both closely regulated biological processes that play a central role in tissue homeostasis, development, and disease. The antiapoptotic protein *Bcl-2* functions not only as an antiapoptotic protein but also as an anti-autophagy protein via its inhibitory interaction with Beclin 1 [80]. Beclin 1 functions in the lysosomal degradation pathway of autophagy and induces autophagic cell death in cancer cells. *Bcl-2*/*Bcl-xL* can bind Beclin 1 and inhibit Beclin 1-dependent autophagic cell death in cancer cells. The BH3 domain of Beclin 1 also binds to the BH3 binding groove of *Bcl-xL*. When the BH3 binding groove of *Bcl-xL* is mutated, the interaction between *Bcl-xL* and Beclin 1 is disrupted. Furthermore, the interaction between Beclin 1 and *Bcl-xL* can be inhibited by ABT737 at low doses, which stimulates autophagy without inducing cell apoptosis. A novel Bcl-xL inhibitor, Z36, efficiently induces autophagic cell death in HeLa cells. Z36 can competitively inhibit the interaction between *Bcl-xL* and Beclin 1 *in vitro*, and thus it is likely that Z36 induces autophagy by blocking the interaction between *Bcl-xL*/*Bcl-2* and Beclin 1 [41]. *Bcl-2* phosphorylation resulted in increased dissociation of the Bcl-2-Beclin 1 complex and increased Beclin 1-dependent autophagy. The activation of JNK upregulated Beclin 1 expression and mediated *Bcl-2* phosphorylation, thereby promoting cellular survival.

#### 9.2.4. Beclin 1

*Metformin*, a widely used antidiabetic agent, should potentially induce autophagy as an activator of AMPK and an inhibitor of mTOR. *Metformin*'s action is mainly mediated by AMPK activation. 2-Deoxyglucose (2DG) is an inhibitor of glucose metabolism, since it inhibits hexokinase, the first rate-limiting enzyme of glycolysis. Treatment with 2DG leads to intracellular ATP depletion and induces autophagy in prostate cancer cells. *Metformin* inhibits 2DG-induced autophagy, reduces Beclin 1 expression, and triggers a switch from a survival process to cell death [81], suggesting that, depending on cell type, activation of AMPK is not automatically associated with the induction of autophagy.

#### 9.2.5. Chemokines

The CC chemokine, *CCL2* (MCP-1), is one of the most frequently observed chemokines in the microenvironment of tumors. *CCL2* has been demonstrated to play a significant role in prostate cancer neoplasia and invasion. *CCL2* protects prostate cancer PC3 cells from autophagic death via the PI3K/Akt/survivin pathway and showed survivin to be a critical molecule in this survival mechanism [82]. *CCL2* stimulation of PC3 cells, upon serum deprivation, results in Akt hyperphosphorylation of the two key regulatory sites (Ser^473^ and Thr^308^) required for its full activation. Survivin is a 16.5-kDa protein that belongs to the inhibitor of apoptosis protein (IAP) family that functions in mitotic progression and antagonizes caspases, thereby inhibiting apoptosis. Survivin is highly expressed in cancer tissues and its high level of expression is associated with poor prognosis and survival in many cancer types. *CCL2*-mediated survivin upregulation was shown to be PI3K/Akt-dependent; inhibition of this pathway not only abrogated survivin expression but also dramatically reduced cell survival. Strong evidence for interaction between survivin and LC3 was detected by co-immunoprecipitation experiments [82]. As LC3 plays an important role in the formation and expansion of autophagosomal membranes, the survivin-LC3 interaction could inhibit this process, which is then reflected in changes in LC3 localization as observed when starved cells are treated with *CCL2*.

#### 9.2.6. Golgi-Associated Agents

Chemical inhibition of autophagy by incubations with *monensin* (coban) significantly increases the extent of apoptosis, which takes place via the mitochondrial pathway, and shortens the time in which the apoptotic markers are detectable. *Monensin* causes an accumulation of early forms of autophagic vacuoles and blocks the swelling of lysosomes seen in the presence of methylamine. Incubation with *monensin* at higher concentrations (10 and 100 *μ*M) resulted in severe mitochondrial damage and marked dilatation of the Golgi apparatus and rough ER cisternae [83]. Interruption of microtubules with microtubule destabilizing *nocodazole* impairs the conversion of LC3-I to LC3-II but does not block the degradation of LC3-II-associated autophagosomes. Strong inhibition of autophagic vacuole accumulation was found in *nocodazole*-arrested pseudoprometaphase cells. The loss of microtubules induced by *nocodazole* treatment results in the disconnection of Golgi stacks and dispersal into 70–100 smaller fragments [84].

#### 9.2.7. Heat Shock Proteins

Heat shock protein 90 (Hsp90) forms a protein complex to maintain the stability of its client proteins. Disruption of this protein complex with specific Hsp90 inhibitors leads to proteolytic degradation of the client proteins, usually through the ubiquitin-proteasome pathway. Hsp90 forms a complex with Beclin 1 through an evolutionarily conserved domain to maintain the stability of Beclin 1. *Geldanamycin*, an Hsp90 inhibitor, effectively promoted proteasomal degradation of Beclin 1 in a concentration-dependent and time-dependent manner [85]. A small molecule called *2-phenylethynesulfonamide* (synonym, also called pifithrin-*μ*, PES) interacts selectively with Hsp70 and leads to a disruption of the association between Hsp70 and several of its cochaperones and substrate proteins. *PES* was identified as a novel Hsp70 inhibitor. *PES*-mediated inhibition of Hsp70 family proteins in tumor cells results in impairment of the autophagy-lysosome system. *PES* impaired the mitochondrial localization of p53 [86]. The *p14/p19* ^ARF^ tumor suppressor gene is frequently mutated in human cancer. ARF (alternative reading frame) has been shown to localize to mitochondria and to induce autophagy. Treatment of cells with *PES* blocks the trafficking of ARF to mitochondria.

#### 9.2.8. Lysosomal Function

*Bafilomycin A1* is in the plecomacrolide subclass of macrolide antibiotics, and a highly specific inhibitor of V-ATPase, whereas other types (F-type, P-type, Ca^2+^, and K^+^) of ATPases are not affected by this antibiotic. The anticancer effect of *bafilomycin A1* is well known and is attributed mainly to the inhibition of autophagy by preventing the fusion of autophagosomes with dysfunctional lysosomes, consequently triggering apoptosis [87]. *Lucanthone* (miracil D) has been extensively used as an antischistome agent. The drug also blocks topoisomerase II activity and has been reported to inhibit AP (apurinic/apyrimidinic) endonuclease, an important enzyme in DNA base excision repair. *Lucanthone* inhibits autophagy, caused by induction of lysosomal membrane permeabilization. *Lucanthone* is a novel autophagic inhibitor that induces apoptosis via cathepsin D accumulation. In addition, *lucanthone* enhanced the anticancer activity of the histone deacetylase inhibitor vorinostat [88]. *Lucanthone* is currently being investigated as a sensitizer to chemotherapy and radiation due to its ability to interfere with DNA repair. *Chloroquine* (avloclor) is a well-known 4-aminoquinoline class drug that is widely used for prophylaxis treatment against malaria. Pharmacologically, *chloroquine* is a weak base and is trapped in acidic organelles like lysosomes, resulting in increased vacuolar pH6.0. *Chloroquine* and its analog *hydroxychloroquine* (plaquenil) are the only clinically relevant autophagy inhibitors that block lysosyme acidification and degradation of autophagosomes [89]. *Chloroquine*, through its lysosomotropic properties, as well as its autophagy inhibition ability, may be a promising agent to be used in combination with chemotherapeutic agents to improve clinical results.

#### 9.2.9. Small Regulatory RNAs

Small regulatory RNAs have become a specific and powerful tool to turn off the expression of target genes. Their actions include the suppression of overexpressed oncogenes, retarding cell division by interfering with cyclins and related genes or enhancing apoptosis by inhibiting anti-apoptotic genes. Paclitaxel, which stabilizes microtubules and causes apoptosis, offers both symptomatic and survival benefits for lung adenocarcinoma. Both autophagy and apoptosis are induced in cancer cells during the course of paclitaxel treatment. Paclitaxel treatment could lead to the formation of acidic vesicular organelles and the induction of Atg5, Beclin 1, and LC3 expression. Paclitaxel-mediated apoptotic cell death was further potentiated by pretreatment with *Beclin 1 siRNA* [90]. Lysosomal-associated membrane protein 2 (Lamp-2) is a ubiquitous lysosomal membrane protein that is highly expressed in normal human pancreatic tissue and is required for the proper fusion of lysosomes with autophagosomes in the late stage of the autophagic process. Silencing with a specific *Lamp-2 siRNA* abolished immunofluorescence particles for Lamp-2, confirming depletion of Lamp-2 protein. LC3-positive immunofluorescence puncta significantly increased in *Lamp-2*-silenced cells, suggesting that Lamp-2 depletion correlated with the accumulation of autophagosomes and a relative paucity of autolysosomes in pancreatic acinar cells. Lamp-2 depletion causes a “traffic jam” that culminates in the accumulation of autophagosomes [91]. MicroRNAs (miRNAs) are a class of endogenous, 22–24 nucleotide RNA molecules with the ability to induce mRNA degradation, translational repression, or both, via pairing with partially complementary sites in the 3′ UTR of the targeted genes. miRNAs can control the expression of autophagic genes, thereby modulating autophagic activity. Indeed, inhibition of the expression by* beclin 1 miR-30a* leads to suppression of autophagic activity [92].

## 10. Technical Errors in Autophagy Study

One of the technical problems in the study of autophagy is the lack of convenient and reliable methods to detect it. Uncertainty in the confirmation of the fully functional autophagic process could be one of the causes of confusion related to the role of autophagy in cell death. These types of confusion and difficulties are more pronounced in animal experiments that are critical in the study of cancers. LC3 is now widely used to monitor autophagy. One approach is to detect LC3 conversion (LC3-I to LC3-II) by immunoblot analysis because the amount of LC3-II is clearly correlated with the number of autophagosomes. Despite a higher molecular weight than LC3-I, LC3-II migrates more rapidly in SDS-PAGE compared to LC3-I, likely due to its higher hydrophobicity associated with the phosphatidylethanolamine group. However, LC3-II itself is degraded by autophagy, making interpretation of LC3 immunoblotting results problematic. Furthermore, the amount of LC3 at a certain time point does not indicate autophagic flux. Monitoring of the natural autophagic substrate p62 (also called sequestosome 1) has been widely used to assess autophagic flux. The degradation of GFP-LC3 was clearly less sensitive for inducers and activators of autophagy than GFP-p62 [93]. A reduction in the abundance of p62 due to its sequestration into autophagosomes, which results from the direct molecular interaction between LC3 and the LC3 interacting motifs (LIR) of p62, a 22-amino-acid acidic peptide motif, is interpreted as a sign of increased autophagy [94]; however, endogenous p62 is not produced at a constant level because some cellular stress conditions lead to induction of p62 at the transcriptional level, meaning that the level of p62 proteins is not solely determined by its turnover. Earlier autophagy studies relied on cell staining and fluorescent microscopy. In particular, the overexpression of GFP-LC3, in which GFP (green fluorescent protein) is expressed as a fusion protein at the amino terminus of LC3, was widely used to measure autophagy. These studies are limited, however, by several issues as follows: incorporation into protein that aggregates independent of autophagy, induction of autophagy by transfection procedures, sensitivity of GFP-LC3 to acid pH, and cessation of fluorescence [95]. GFP is more resistant than LC3 in response to lysosomal degradation. When analyzing autophagic flux based on GFP-LC3 degradation, the accumulation of free GFP and GFP-LC3-II in the absence and presence of a lysosomal inhibitor should be compared. These may represent a serious problem in experiments testing the autophagic process.

Many compounds to regulate autophagy are limited by the uncertainty as to whether they specifically target one step of the autophagy process. It is of interest to compare relative drug effects obtained under different settings, including conditions, time points, and concentrations. The compound 3-MA has been touted as an autophagy inhibitor but requires mM concentrations to inhibit class III PI3Ks involved in autophagy. 3-MA not only arrests autophagy but also prevents apoptosis by inhibiting the release of cytochrome *c* and cathepsin B. Rapamycin, commonly applied to induce autophagy, enhanced autophagy under full medium but had a surprising inhibitory effect under nutrient deprivation conditions. Bafilomycin A1 is also commonly employed to suppress autophagic flux due to its inhibition of the vacuolar type H^+^-ATPase (V-ATPase), blocking acidification of lysosomes and endosomes. However, long-term treatment (>4 h) of cells with bafilomycin A1 also interferes with the trafficking of proteosomes, endosomes, and other cellular processes [96]. Therefore, a need exists for chemicals that target specific components of the autophagy machinery for use as research tools, to address questions about autophagy mechanisms and investigate the role of autophagy in cancers.

## 11. Concluding Remarks

In this paper, the molecular players and mechanisms associated with autophagy have been described. Although autophagy is an important mechanism used by tumor cells to tolerate metabolic stress and is involved in nearly all stages of tumorigenesis and tumor progression, the issue of whether autophagy is aimed primarily at cell survival or at cell death is likely to remain a subject of debate for some time. As autophagy is a dynamic process that is difficult to measure and quantify, new techniques for assessing autophagy need to be developed to ensure progress in this area of investigation. The multifaceted nature of autophagy and its diverse cross-talk with other biological processes must be carefully considered when the autophagic system is targeted for anticancer benefit.

## Figures and Tables

**Table 1:** Proautophagics.

Class (target)	Compounds	Autophagic mechanisms	Reference
Beclin 1	ABT-737 Fenretinide Xestospongin B Okadaic acid	Disruption of Bcl-2/Bcl-xL binding to Beclin 1 Increase in Beclin 1 expression Disruption of the complex formed by IP_3_R and Beclin 1 Increase in Beclin 1 pathways	[52] [53] [54] [55]

DNA damage	UV ROS Camptothecin	p53 activation PTEN upregulation mTOR suppression	[56]

ER stressors	A23187 Thapsigargin Tunicamycin Brefeldin A Bortezomib Sorafenib	GRP78 induction Increase of Ca^2+^ in cytosol PERK/elF2*α* pathway Induction of GRP78 and GRP94 Unfolded protein response Inhibition of mTOR signaling	[57] [58] [59]

Farnesyltransferase inhibitors	Manumycin A FTI-276 Lonafarnib	Akt downregulation and mTOR phosphorylation	[60]

Golgi-associated agents	Brefeldin A Paclitaxel	Interruption of trafficking of N-linked glycoproteins Fragmentation of Golgi apparatus	[61] [62]

HDAC inhibitors	SAHA Valproic acid FK228	Downregulation of Akt and mTOR activity Increase in mitochondrial ROS Nuclear translocation of AIF	[63] [64] [65] [66]

Mitochondrial agents	Selenite Resveratrol	Degradation of mitochondrial proteins Inhibition of mitochondrial function	[7] [67]

mTOR inhibitors	Rapamycin Berberine Torin1 Ku-0063794 PM02734	mTORC1 inhibition Akt suppression and p38 MAPK upregulation Inhibition of mTORC1 and mTORC2 Dephosphorylation of mTORC1 substrate 4E-BP1 DAPK activation	[68] [69] [70] [71] [72]

Pathogens	Bacteria HSV-1	Ubiquitin-p62-NDP52 pathway PKR and eIF2*α* activation	[73] [74]

Proteasome inhibitors	MG-132 Glidobactin A	ER stress induced by misfolded proteins Catalytic subunits of 20S proteasome	[75] [76]

**Table 2:** Antiautophagics.

Class (target)	Compounds	Antiautophagic mechanisms	Reference
Atg proteins	Calpain-1, -2 Caspase-3, -6, -8	Defect in autophagic process and apoptotic enhancement	[77]

Autophagosome formation	Verteporfin 3-Methyladenine	Inhibition of autophagic degradationInhibition of class III PI3K activity	[78] [79]

Bcl-2 family	Bcl-2/Bcl-xL	Inhibitory interaction with Beclin 1	[80] [41]

Beclin 1	Metformin	Decrease in Beclin 1 expression and AMPK activation	[81]

Chemokines	CCL2	Survivin upregulation	[82]

Golgi-associated agents	Monensin Nocodazole	Dilatation of Golgi apparatus Disconnection of Golgi stacks and dispersal into fragments	[83] [84]

Heat shock proteins	Geldanamycin PES	Promotion of Beclin 1 degradation Impairment of the autophagy-lysosome system	[85] [86]

Lysosomal function	Bafilomycin A1 Lucanthone Chloroquine	Prevention of fusion of autophagosomes with lysosomes Lysosomal membrane permeabilization Blocking of lysosyme acidification	[87] [88] [89]

Small regulatory RNAs	Beclin 1 Lamp-2 mi-30a	Potentiation of apoptotic death, autophagosome degradation Impairment of autolysosome formation Beclin 1 translational suppression	[90] [91] [92]
